# Supplementary material for: The Role of miR-21 in Osteoblasts–Osteoclasts Coupling In Vitro
Source: Cells. 2020 Feb 19;9(2):479. doi: 10.3390/cells9020479 (PMC7072787; doi:10.3390/cells9020479)
Supplement: Supplementary file 1 [file cells-09-00479-s001.pdf]

Table 1 S. Sequences of the primers used in the experiment.

Gene name	Gene abbreviation	Primer	Sequence 5'-3'	Amplicon lenght [bp]	Annealing temperature [°C]	Accesion no.
Osteopontin	*Opn*	F:<br>R:	AGACCATGCAGAGAGCGAG<br>GCCCTTTCCGTTGTTGTCCT	340	57,3	NM_001204203.1
Osteocalcin	*Ocl*	F:<br>R:	GGTGCAGACCTAGCAGACACCA<br>CGCTGGGCTTGGCATCTGTAA	100	57	NM_001032298.3
Collagen type I	*Coll-1*	F:<br>R:	CAGGGTATTGCTGGACAACGTG<br>GGACCTTGTTTGCCAGGTTCA	107	61,4	NM_007742.4
Tartrate-resistant acid phosphatase	*Trap*	F:<br>R:	GTCTCTGGGGGACAATTTCTACT<br>GTTTGTACGTGGAATTTTGAAGC	241	60	XM_006509945.3
Runt related transcription factor 2	*Runx-2*	F:<br>R:	TCCGAAATGCCTCTGCTGTT<br>GCCACTTGGGGAGGATTTGT	130	58,8	NM_001271630.1
Receptor activator of nuclear factor kappa B	*Rank*	F:<br>R:	TTAAGCCAGTGTTTCACCGG<br>ACATACACCACGATGATGTC	473	58,8	NM_009399.3
Receptor activator of nuclear factor kappa B ligand	*Rankl*	F:<br>R:	ACGCAGATTTGCAGGACTCGAC<br>TTCGTGCTCCCTCCTTTCATC	493	58,8	NM_011613.3
Osteoprotegerin	*Opg*	F:<br>R:	AGCCACGCAAAAGTGTGGAA<br>TCCTCTCTACACTCTCGGCA	149	58,8	NM_008764.3
Cathepsin K	*Ctsk*	F:<br>R:	TAACAGCAAGGTGGATGAAATCT<br>CTGTAGGATCGAGAGGGAGGTAT	195	60	NM_007802.4
Carbonic anhydrase II	*Ca II*	F:<br>R:	TCAGGGAGCCCATTACTGTC<br>TCCAAATCACCCAGCCTAAC	234	60	NM_001357334.1
Matrix metalloproteinase 9	*Mmp-9*	F:<br>R:	TTGCCCCTACTGGAAGGTATTAT<br>GAGAATCTCTGAGCAATCCTTGA	172	60	NM_013599.4
Glyceraldehyde-3-phosphate dehydrogenase	*Gapdh*	F:<br>R:	TGCACCACCAACTGCTTAG<br>GGATGCAGGGATGATGTTC	177	60	XM_017321385.2
B cell leukemia/lymphoma 2	*Bcl-2*	F:<br>R:	GGATCCAGGATAACGGAGGC<br>ATGCACCCAGAGTGATGCAG	141	58,8	NM_009741.5
BCL2-associated X protein	*Bax*	F:<br>R:	AGGATGCGTCCACCAAGAAGC<br>GGTTCTGATCAGCTCGGGCA	251	58,8	XM_011250780.3

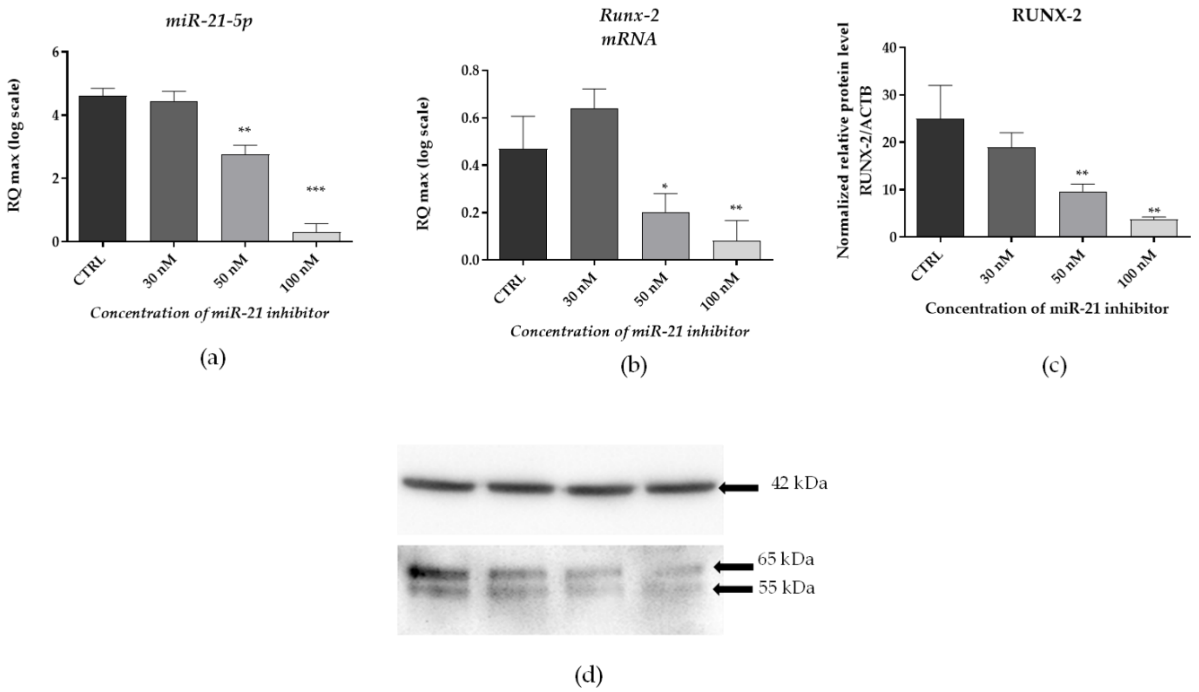

Figure 1S. Analysis of effectiveness of miR-21 inhibition in MC3T3 cell line. The analysis included determination of miR-21 levels using Two-tailed RT-qPCR (a) and determination of target gene expression, *Runx-2* using RT-qPCR (b). The RUNX-2 protein was determined using Western blot technique. The highest effectives of miR-21 inhibition was noted in cultures were 100 nM concertation was applied (~80% of effectiveness). However, based on cells viability for further assays 50 nM concertation was used. The effectiveness of 50 nM miR-21 inhibition determined based on miR-21 levels and Runx-2 expression (mRNA and protein level) was around 50%.

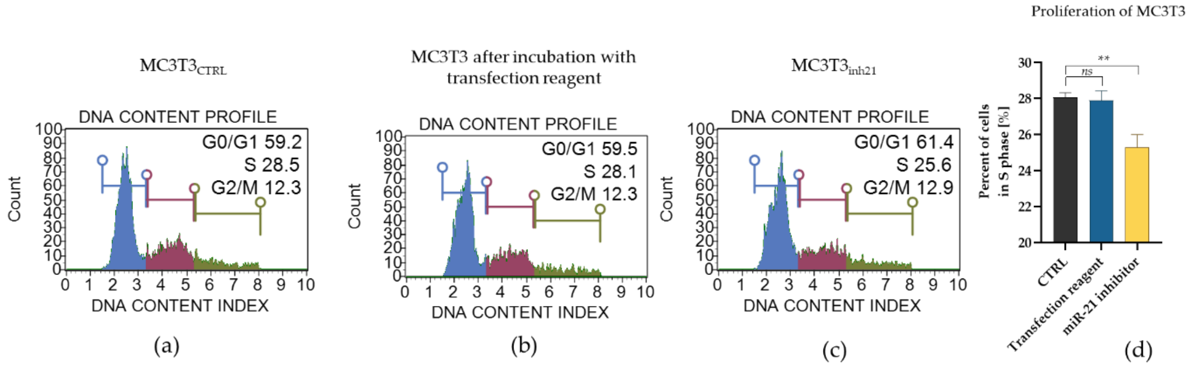

Figure 2S. The influence of transfection with miR21 inhibitor on MC3T3 proliferative activity. Representative histograms indicating the distribution of pre-osteoblasts in the cell cycle (a-c). The percentage of cells in S phase was compared and statistical analysis was performed using One-way analysis of variance and Dunnett's post hoc test (d). The data were analysed using GraphPad Software (Prism 8.20, CA, USA). Differences with a probability of $p<0.05$ were considered as significant. The significant differences are indicated with asterisks (** $p<0.01$ and *** $p<0.001$), while non-significant differences are marked as *ns*.

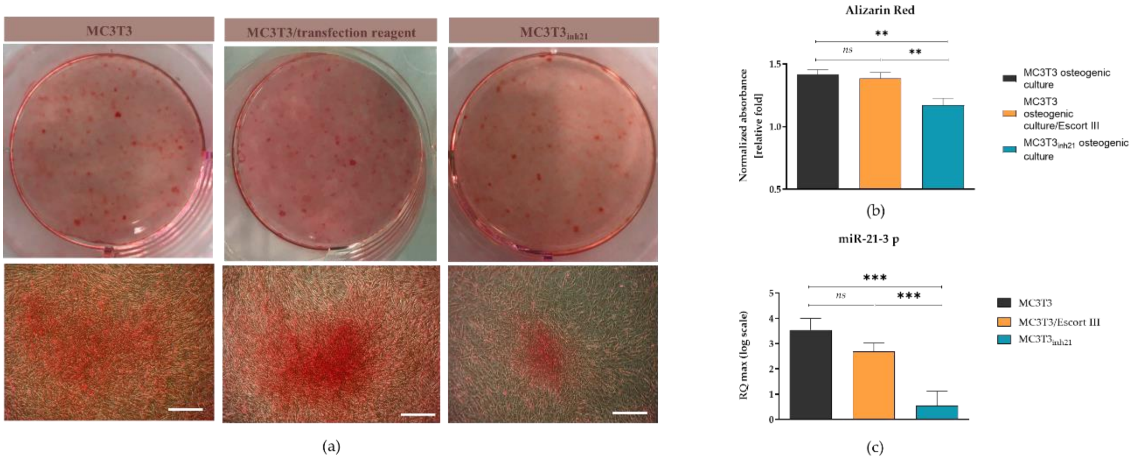

Figure 3S. The results showing a specific action of miR-21 inhibitor in osteogenic cultures. Analysis included Alizarin Red staining of extracellular matrix (a) and measurement of staining insensitivity (b). Moreover, miR-21 levels were determined using Two-tailed RT-qPCR (c). The osteogenic potential of MC3T3 transfected with miR-21 was decreased, what was correlated with lowered mineralization of extracellular matrix (a-b). Osteogenic capability of MC3T3 and miR-21 levels were not influenced by transfection procedure (a-c). Statistical analysis was performed to determine significance of obtained results. The significant differences are indicated with asterisks (** $p < 0.01$ and *** $p < 0.001$), while non-significant differences are marked as *ns*.

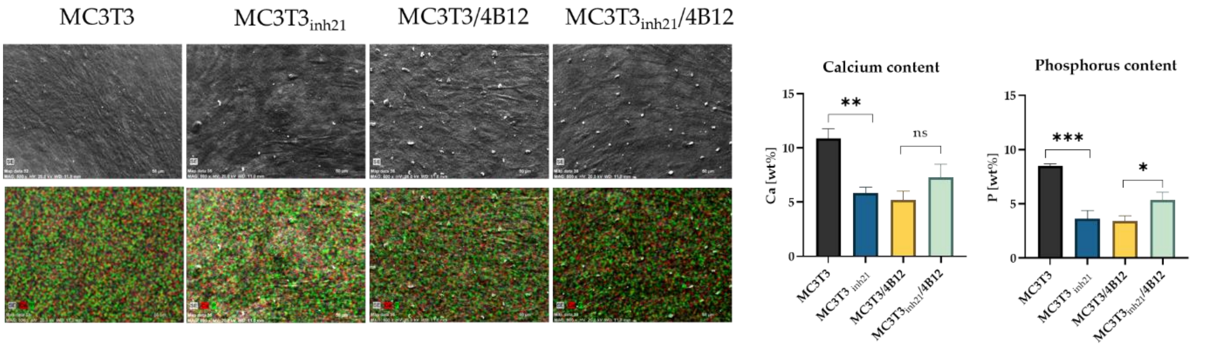

Figure 4S. The analysis of calcium (Ca) and phosphorus (P) content deposited in extracellular matrix formed by MC3T3. Measurements were performed using SEM-EDX. Images of cultures were taken under 500-fold magnification. SEM-EDX maps indicate on Ca and P distribution. The elements were measured and their content was presented as weight percentage (wt%). Statistical analysis was performed to determine significance of obtained results. The significant differences are indicated with asterisks (** $p < 0.01$ and *** $p < 0.001$), while non-significant differences are marked as *ns*.

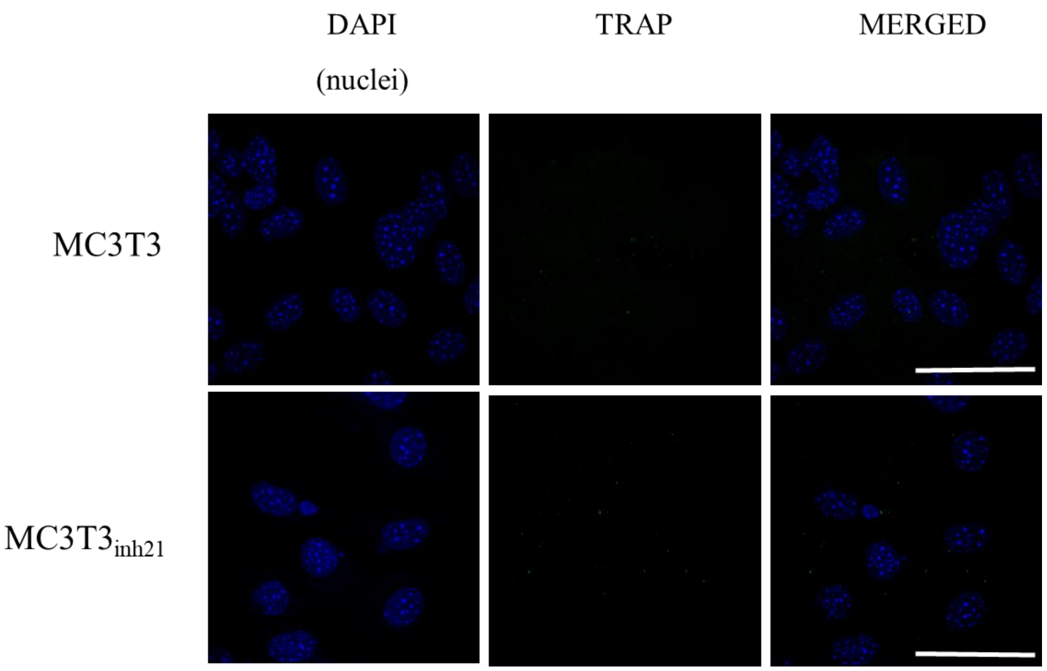

Figure 5S. The influence of miR-21 inhibition on TRAP expression in the MC3T3 osteoblasts. Representative images showing co-localization of TRAP (green signal) with nuclei (blue, DAPI stained) in pre-osteoblast cultures, both control (MC3T3) and with decreased expression of miR-21 ($MC3T3_{inh21}$). The images were taken under 60-fold magnification. The scale bar is equal 50 μm.
